# Using a Value Chain Approach to Map the Pig Production System in Rwanda, Its Governance, and Sanitary Risks

**DOI:** 10.3389/fvets.2021.720553

**Published:** 2022-01-18

**Authors:** Anselme Shyaka, Rupert J. Quinnell, Nadine Rujeni, Eric M. Fèvre

**Affiliations:** ^1^Faculty of Biological Sciences, School of Biology, University of Leeds, Leeds, United Kingdom; ^2^School of Veterinary Medicine, College of Agriculture, Animal Sciences and Veterinary Medicine, University of Rwanda, Nyagatare, Rwanda; ^3^Center for One Health, University of Global Health Equity, Kigali, Rwanda; ^4^School of Health Sciences, College of Medicine and Health Sciences, University of Rwanda, Kigali, Rwanda; ^5^Institute of Infection, Veterinary and Ecological Sciences, University of Liverpool, Leeds, United Kingdom; ^6^International Livestock Research Institute, Nairobi, Kenya

**Keywords:** pig, Rwanda, value chain analysis, value chain governance, sanitary risks, cysticercosis

## Abstract

Rwanda has a fast growing pig production sector projected to continue expansion, due to rising local and regional demand. We undertook a value chain analysis to establish the flows of pigs and pork in Rwanda and the roles of various actors involved, and to understand governance and sanitary risks in the value chain. Cross-sectional qualitative data were collected through focus group discussions and key informant interviews with farmers, brokers, butchers, abattoir managers, and veterinarians. Data were collected on pig production methods and inputs, the source and destination of live and slaughtered pigs, value-adding infrastructures (abattoirs and processing factories), the people involved and interactions between them, governance, and challenges. Pig production in Rwanda is dominated by smallholders, mainly as a source of supplementary income and secondarily for manure. Emerging medium-sized and large pig farms were also identified, located mainly around urban areas. Live pig markets are the main mechanism allowing various actors to buy/sell pigs. Brokers have an important role in pig transactions: they are key in setting prices at markets, examining pigs for disease, organising the supply of pigs for abattoirs and for export. Only a few formal pig abattoirs were identified, which mainly supply to pork processing factories based in Kigali and/or export to customers. Local consumers rely on informal slaughtering at farm or bar/restaurant backyards, with irregular veterinary inspection. Formal abattoirs were attended by a veterinary inspector, however a lack of record keeping was noted. Sanitary risks identified were a lack of biosecurity throughout the chain and poor hygiene at slaughter places. Lingual palpation was practised in pig markets to identify cysticercosis infection, however cyst-positive pigs were not destroyed, but were sold for reduced prices in the same market or later informally sold by the owner. There are few veterinarians attending farms, with most services provided by less qualified technicians or self-treatment of pigs by farmers. Overall, this production system is characterised by a high degree of informality at all nodes, combined with the rapid growth trajectory in the sector. These findings provide a basis to plan interventions tailored to vulnerabilities identified in the Rwanda pig value chain.

## Introduction

The total demand for animal products in developing countries is expected to double by 2030 and pigs and poultry are expected to dominate meat supply compared to ruminants (1). Similarly, pig production in Rwanda is increasing rapidly to meet rising demand in urban and rural areas, and for export to neighbouring countries (2). The pig population in Rwanda is currently estimated at 1.8 million pigs (3), largely in smallholder production; in some regions of the country, 80% of households are estimated to keep pigs, with 1–2 grown pigs per household. Pigs contribute about 21% of total dressed meat in Rwanda, compared to 46% from cattle (3). However, pig meat production is expected to increase to meet nutritional requirements of the growing Rwandan population, and to increase household incomes in rural areas. To help stimulate pig production, the Ministry of Agriculture and Animal Resources set out a plan to distribute 1.25 million pigs from 2018 to 2024. Total pork production was projected to rise by about 40%, from 19,869 tonnes in 2017 to 27,871 tonnes in 2022 (3).

Pig production in tropical Africa is challenged by various factors related to source and quality of feeds, animal genetics/breeding, and veterinary services (3–8). Moreover, with more pig production there is also the potential for an increased risk of both porcine and human diseases. Of particular concern is the potential for an increased burden of human cysticercosis, which is now recognised as the most important food-borne parasitic disease (9, 10). Addressing these issues requires information on the organisation and structure of local pig production systems.

Value chain analysis is a useful method for understanding livestock production systems. Value chain mapping describes how people manage livestock and their products, and identifies key actors, their linkages, and relative influence. Such mapping can also establish fundamentals for food safety risk analysis which in turn offers an opportunity to design interventions. Here, we carried out a value chain analysis of pig production in Rwanda, focusing on mapping and governance, two of the four components making up the value chain analysis as defined by Kaplinski and Morris (11). Mapping helped to establish the profiles of people involved in the flows of pigs and pig products as well as associated processes. We studied governance to examine the relative influence of different people and groups and the formal and informal rules governing the value chains. The objectives were to: (1) Identify existing infrastructures in the pig value chain including: pig markets, slaughterhouses/abattoirs, pork processing factories, feed manufacturing companies, veterinary clinics, etc., (2) Define and characterise the various value chains supplying pigs and pork in Rwanda and the proportions of product reaching consumers through each chain, (3) Identify the main actors directly involved in the flow of pigs and pork and the interactions between them, (4) Characterise the governance and food safety risks in the Rwanda pig value chain.

## Materials and Methods

A cross-sectional study was conducted in all provinces of Rwanda between August 2017 and September 2018. Broadly, we followed the methodology applied in similar studies in Kenya (12, 13).

### Study Area and Selection of Study Participants

**Figure 1:**
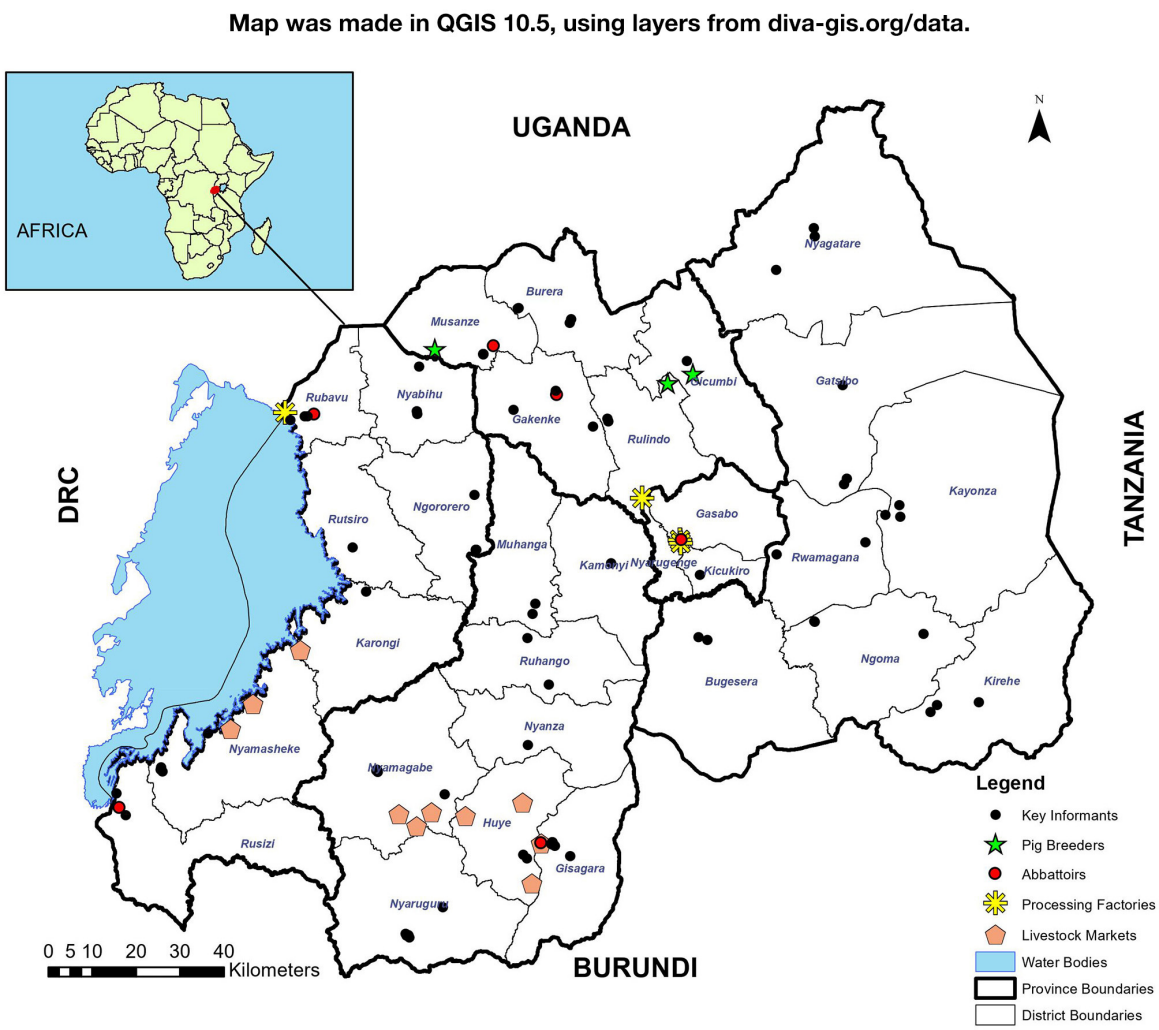
Map of Rwanda with administrative boundaries showing the various study sites.

Interviews were conducted in all provinces of Rwanda (Figure 1) in the form of focus group discussions (FGDs) and key informant interviews (KIIs) (Table 1).

**Table 1:** Number of focus group discussions (FGD) and key informant interviews (KII) carried out and number of participants in each.

**Province**	**FGD (Total Participants)**	**KIIs (No.)**
Kigali	2 FGD with Pork suppliers (16)	DVO (2), Farmers (19)
Southern	7 FGD with Farmers (56), 5 FGD with Traders/Brokers (30), 4 FGD with Butchers (28)	DVO (6), SVO (3), Farmers (26), Pork vendors (2)
Northern	2 FGD with Farmers (12), 2 FGD with Brokers (12)	DVO (5), SVO (1), Abattoir managers (2), Farmers (11), President of cooperative (1)
Western	–	DVO (5), Farmers (12), Brokers (2), Abattoir managers (4)
Eastern	–	DVO (2), Farmers (20)

Initial interviews and conversations with district veterinary officers (DVOs) and the representative of the Rwanda Pig Farmers' Association allowed us to identify existing structures in the value chain such as markets, abattoirs, and key pig farmers. We were then able to snowball from these initial conversations to reach further stakeholders in the value chains. Snowball sampling is a technique in which identified study participants recruit future study subjects from among their peers. Despite the method being non-probabilistic, it is recommended for the recruitment of hard-to-reach stakeholders or when there is no prior knowledge about the study subjects (21, 22).

At these places, interviews were conducted with key informants such as managers (at abattoirs) or sector veterinarians (of the administrative entity where a market and/or abattoir was found) to identify profiles of various people interacting in the value chain and determine origin and destination of pig and pork. Moreover, at these establishments, FGDs were conducted with pig farmers and traders. Where possible, for each category of actors, a FGD was organised and comprised six to eight persons of both genders when possible. Focus group discussions were conducted with farmers, brokers, and butchers. The interviews were conducted in the Kinyarwanda language, which can be spoken by all Rwandans, and data were transcribed in English.

The data gathered through FGDs were supplemented with semi-structured interviews with key informants, who are people with specific knowledge of the site and flow of operations in the value chain. Individual interviews were also conducted with farmers in localities were pig production was not common. Thus, KIIs were done with DVOs, presidents of cooperatives, farmers, and abattoir managers. In total, 277 people (184 men, 93 women) contributed to the data collection, 154 in FGDs, and 123 in KIIs (Table 1).

### Data Collection

The purpose of the research was explained to potential participants prior to the beginning of any interview or discussion. The researchers explained that participation was voluntary and that participants could withdraw at any time without providing an explanation. Where audio recordings were made, permission was sought after explaining the purpose of recording which was to optimise the time and accuracy of data collection. Participants were given a small compensation fee to cover their time and/or travel to the meeting venue. Written consent was obtained from all participants prior to the FGDs and KIIs.

Focus group discussions were conducted using a semi-structured guide and methodology applied in similar studies in Kenya (12–14, 23, 24). Briefly, participants were requested to describe:

- Type of people they interact with in their business, how each category is identified, terms, and incentives that influence working with each category.
- Source of inputs (weaned piglets, grown pigs, feed, water, veterinary services, etc.) and organisation associated with input supply. Clarity was sought on terms and conditions for working with each type, conflicts that may arise, seasonal variation, etc.
- Type of output from their establishment and type of people associated with each type of output. Respondents were asked to characterise the possible buyers, intermediaries, and conditions for buying (existence of contract, incentives, and conditions associated with a type of buyer).
- Government officials they work with, and why or why not.
- How they dispose of sick and/or dead animals, management of other waste from their facility.
- If there are any large companies that interact with them, terms and conditions for their interaction.
- If there is an individual, organisation, or company that dominates the pig market in Rwanda and why they think they are dominant.
- Challenges in doing their work, incentives, and what can be improved in their daily activities and finally their future plans (what they think their business growth will be).

During the KIIs, the participants were asked to:

- Describe the type of people they interact with in their business, the main groups of livestock holders in their area, their organisation as well as the quality and seasonality of livestock products associated with each category.
- Describe available organised systems by which livestock holders obtain their pigs and feed, or through which farmers sell their pigs and pig products, as well as available transport systems. In addition, they were asked how they handle sick animals and what veterinary services are available in their area.
- Describe their impression of who dominates the pig market and the reasons on which they base this.
- Describe changes that they foresee in the future of the pig production industry.

Most FGDs and KIIs were audio-recorded; when meetings were not recorded, detailed notes were made during the meeting.

Value-chain mapping focuses on understanding the flows of products from source to market, and we did not consider other factors such as animal welfare conditions in this study.

### Data Analysis and Validation

Audio recordings were carefully transcribed to word-processing documents pre-designed to record data under specific sections related to the research questions and emerging themes. When necessary, these recordings were complemented with notes, reports, observations, and flow charts made during interviews. This first step allowed us to create a working structure, which gathered qualitative information collected from different sources.

**Figure 2:**
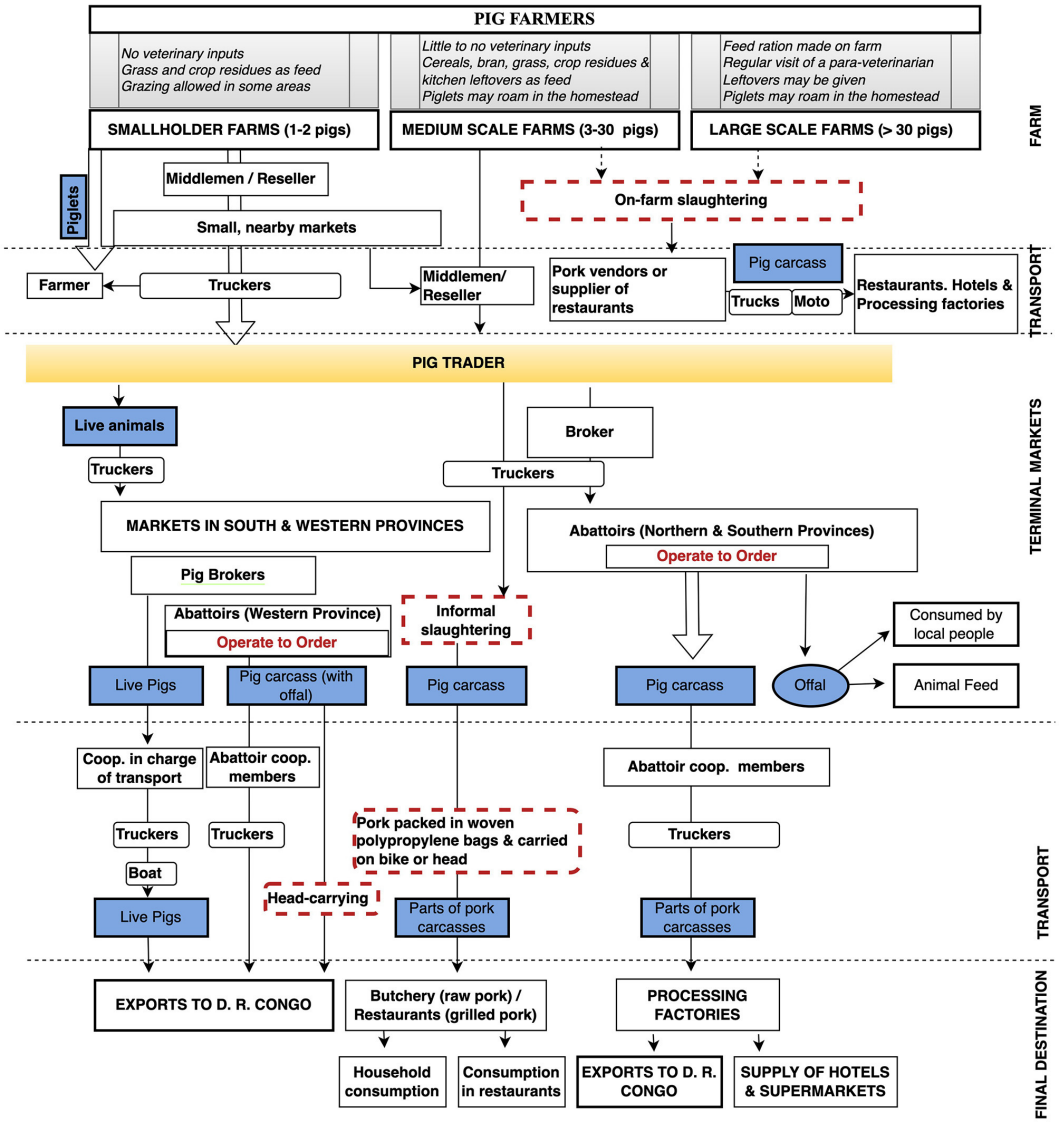
The overall structure of the Rwanda pig value chain. The flow of live pigs and pork in Rwanda. Large arrows indicate more important pathways while dotted red boxes highlight some sanitary risks in the value chain.

**Figure 3:**
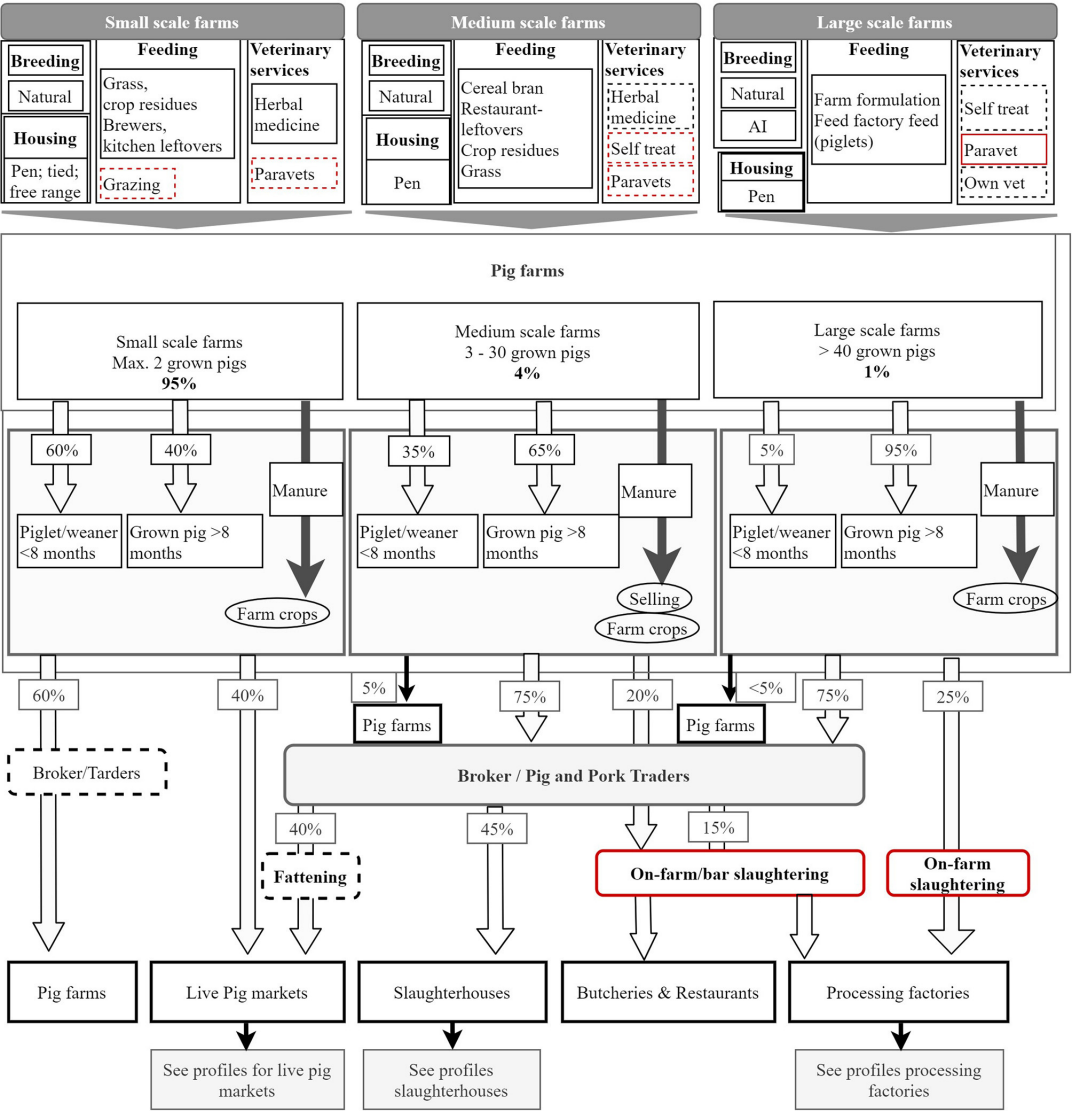
Profile of Rwanda pig farms. Flowchart indicates origins and destination of pigs depending on type of farm. The characteristics of each farming system are summarised at the top and the approximate percentage of each pig category sold is indicated. Red boxes indicate sanitary risks and dotted boxes show occasional flow through.

**Figure 4:**
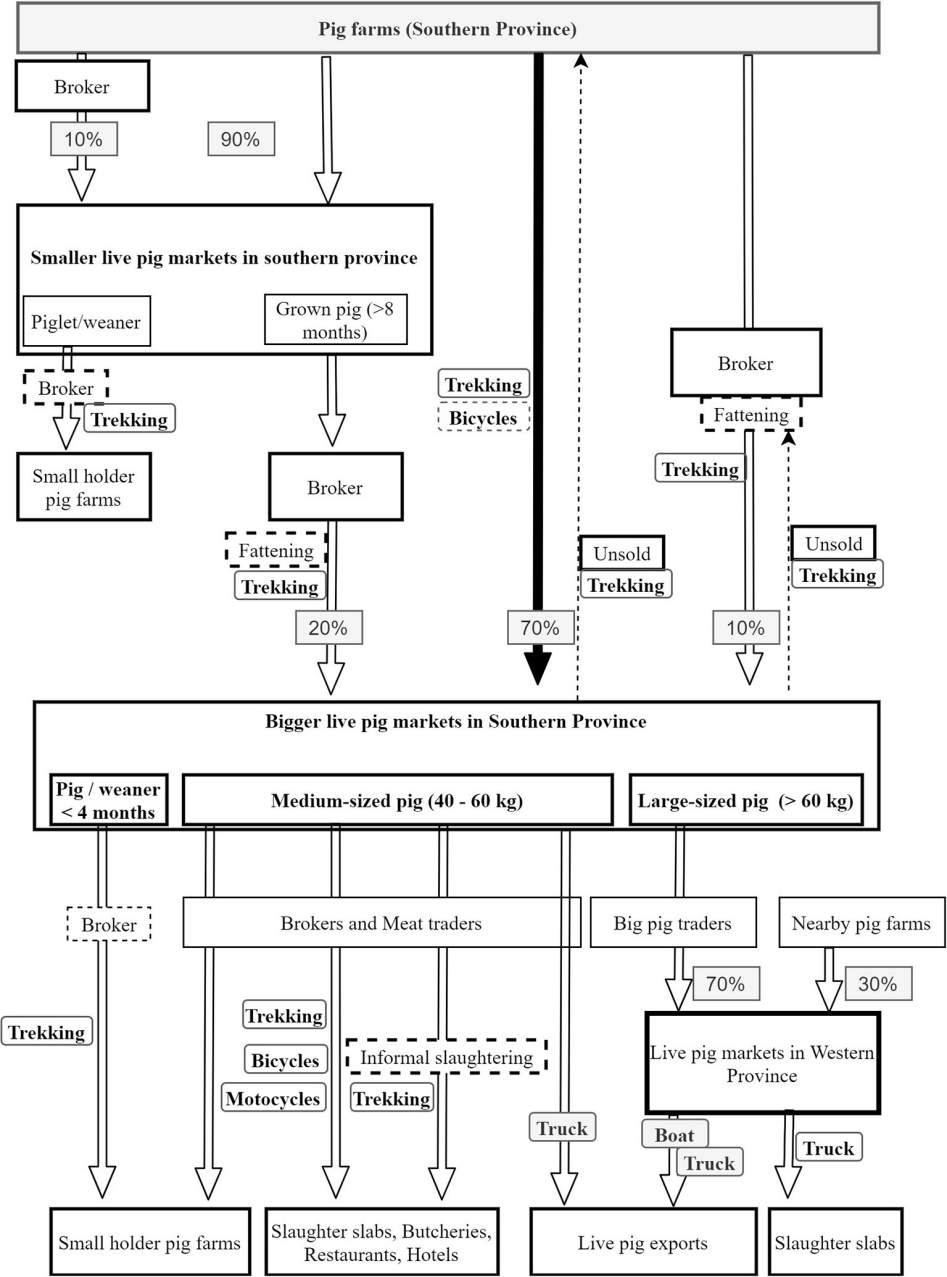
Profile of Rwanda live pig markets. Flowchart indicates origins and destination of live pigs. Of note, live pig markets are found in Southern and Western provinces of Rwanda. The pigs from other provinces have their characteristic flows as explained in the pig farms profile.

**Figure 5:**
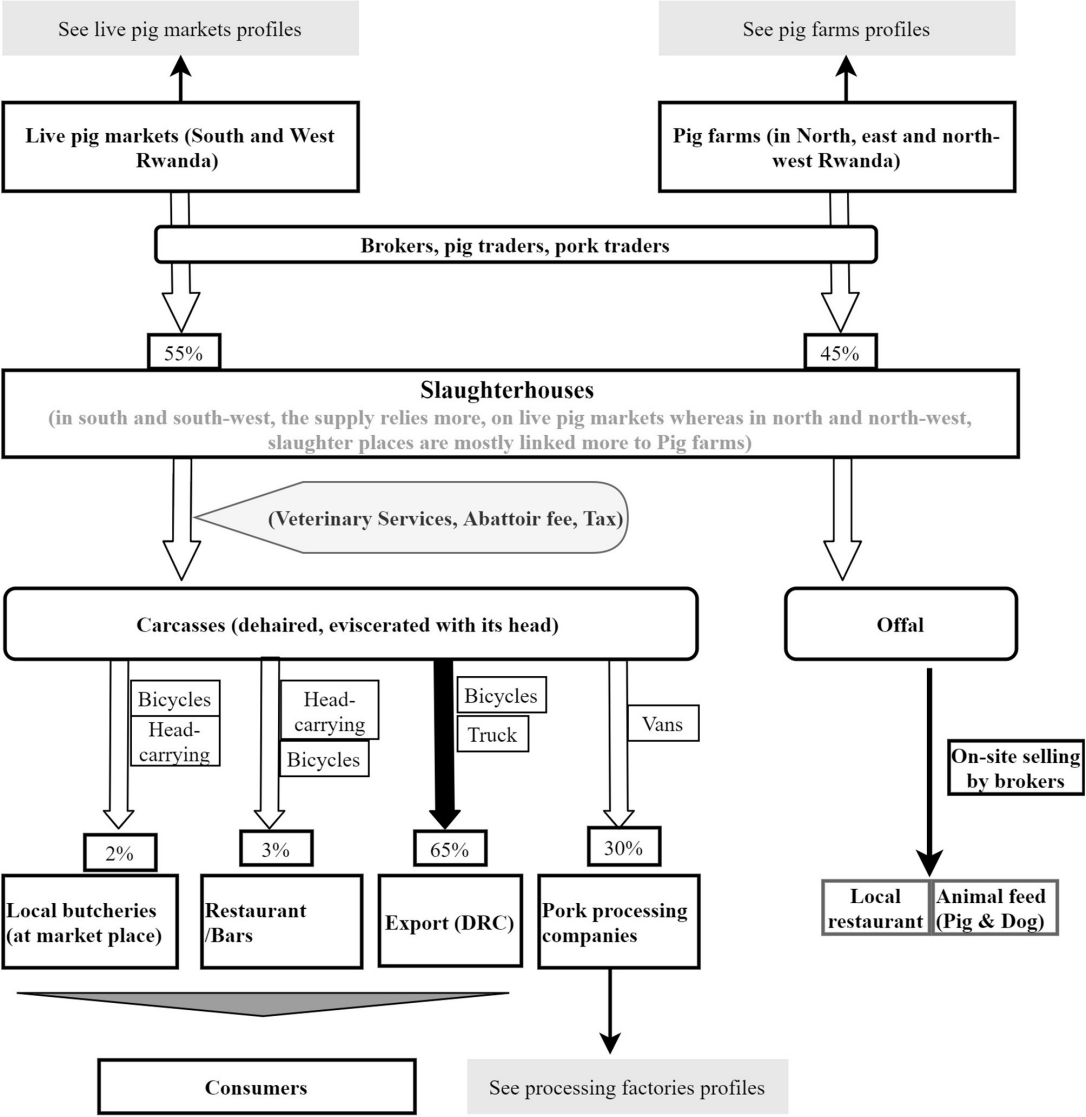
Profile of Rwanda pig slaughter slabs. The flowchart shows sources and destinations of pigs and pig meat. Percentages relate to the volume of pig meat supplied at each segment.

**Figure 6:**
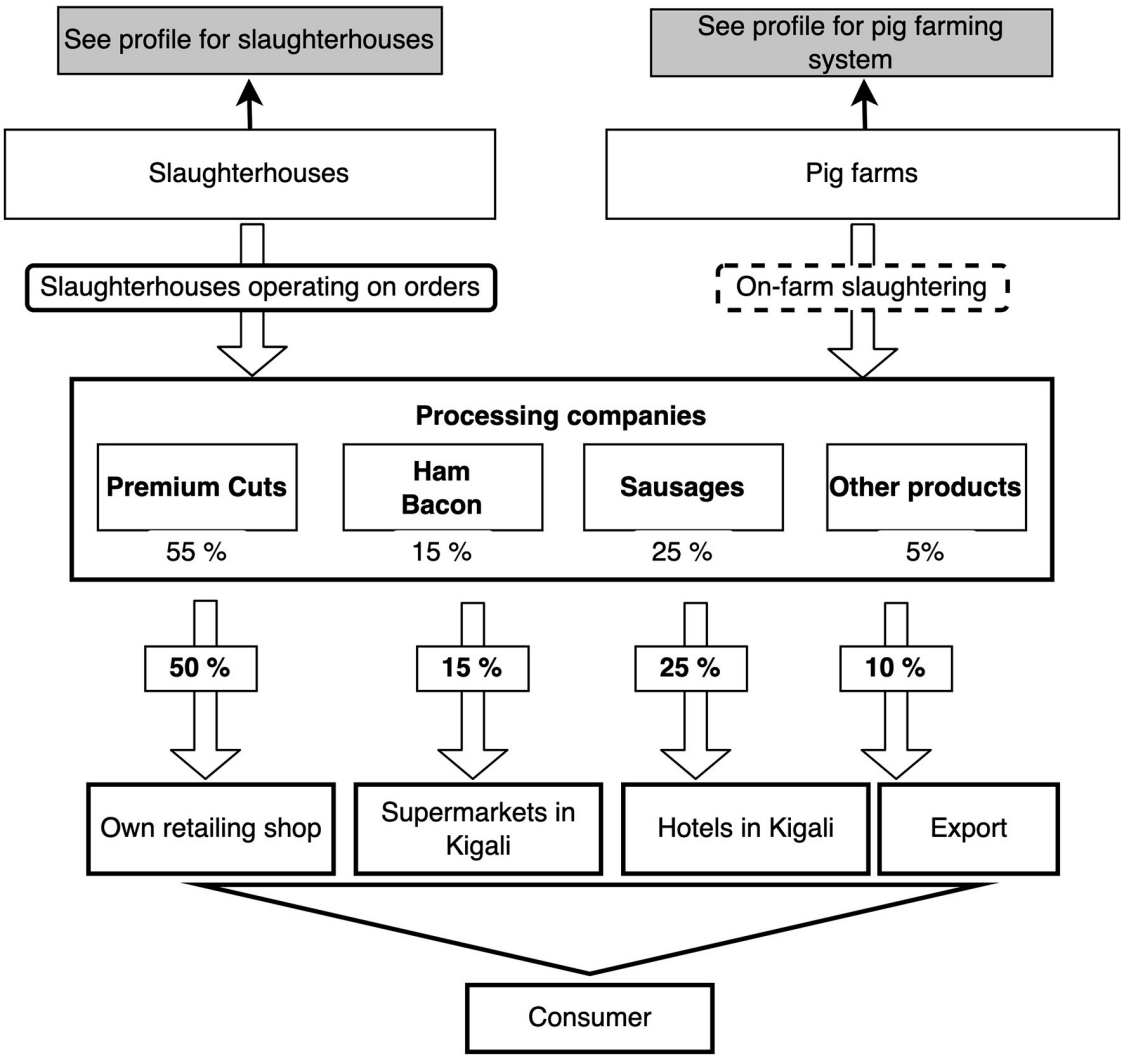
Pork processing company profile: the flowchart shows sources and retailing channels for pork products. Percentages under pork products relate to the ratio of products manufactured. Percentage at the retailing places relate to volume supplied to each venue.

Next, a thematic analysis was conducted to understand the value chain operations, interactions of various actors, flow of live pigs and pig products, source, and supply channels of inputs and finally capture emerging themes and sub-themes. As we used a non-systematic sampling strategy, the data were more suitable for a thematic rather than quantitative analysis (15, 20). This exercise allowed the creation of flowcharts linking various profiles in the value chain (see Figures 2–**6**). To avoid having congested flowcharts, information relating to the source of inputs (feed, breeding, and veterinary services, etc.) was omitted but described in the text. Important nodes in the value chain were linked by arrows to represent the flow of live animals, products or services. Where possible, larger arrows were used to characterise a larger proportional size of the flows.

Information gathered through FGDs was triangulated during KIIs and observations during our visits to various value chain places such as markets, abattoirs, farms, etc. Data triangulation was carried by cross-checking data gathered with responses from other informants, or with direct observations, in order to have a more complete understanding or ascertain the accuracy and completeness of the information (25). When gaps and discrepancies were detected, additional discussions with key informants were undertaken to confirm and, if necessary, update available information.

## Results

Four chain profiles were found making up the overall Rwanda pig value chain (Figure 2). These profiles include pig farming systems in Rwanda (Figure 3), live pig markets (Figure 4), slaughter houses (Figure 5), and processing factories (Figure 6). These chain profiles intersect and often involve the same value chain actors, leading to a complex value chain.

We identified both informal and formal pig value supply chains. Formal supply chains are those flowing through infrastructures regulated by inspection, licencing, and taxation. Informal supply chains are those outside this formal regulatory framework. However, these supply chains are interlinked, with various intersections between both supply systems.

### Chain Profiles for the Farming Systems in Rwanda

#### Typology of Pig Farming Systems in Rwanda

Three different pig production systems were identified in various regions of Rwanda (Figure 3).

##### Small-Scale Pig Production

Small-scale pig production, with one or two grown sows, was identified as the predominant system (90–95% of farms) in most of Rwanda. Small-scale pig farmers are typically engaged in smallholder farming in rural areas, and own one or two grown sows or weaners. Smallholders reported engagement in pig farming as a quick income generation activity and as a source of manure for their crop fields. A pig is easier to sell than a cow, as the lower price makes it easier to find buyers, and it has a lower socio-cultural value. Thus, selling a pig can generate income rapidly for payments such as school fees and health insurance when needed. Others reported keeping pigs as a way to generate enough money to be able to keep cattle in the future, an illustration of the livestock ladder (26). This system is characterised by a near-absence of veterinary inputs or purchased feed.

Participants reported that small-scale farmers sourced weaners from other neighbouring small-scale farmers, chosen according to perceived breed quality attributes such as skin coat colour, or based on reputation of the selling farmer. The purchased weaners are fattened for a period of 8–10 months and then sold. After sale, the small-scale farmer buys another weaner of 2–3 months and re-starts the cycle. A smaller proportion of small-scale farmers keep sows, which are mated to a local boar, and specialise in selling piglets, typically at 2–3 months old, to their neighbours or at market. Pigs in this system were housed in pens with timber walls and roof, and floor of soil or timber, or kept tethered in the backyard. In some regions of southern Rwanda, pigs are allowed to graze, tended by children, while in other regions it was common to see piglets roaming inside and around the household. Pigs are fed on leftover household food, crop residues (especially parts of sweet potatoes), grasses, and sorghum beer brewing waste. In some areas, it was reported that a sow with newborn piglets may be given a sorghum porridge to help it “recover” quickly.

Farmers prefer to use live markets for the sale of pigs where these are available (parts of Southern and Western provinces). Both grown pigs and piglets/weaners are sold at market (see section Source of Pigs). Where pig markets are not available, most grown pigs are sold by farmers to brokers or traders who transport pigs to slaughter places or to live pig markets. However, sales of piglets/weaners do also occur at farm level. The general picture was as follows: Weaned piglets were sold by farmers to other farmers without any form of contract or other formal rules. Some farmers were reportedly known to have a perceived “high breed” (for example, a sow that gives birth to many piglets) and were preferred sellers. In this case, buyers may even pay to “book” a piglet in advance. These farmer-to-farmer transactions generally do not involve a third-party. However, on a few occasions, it was reported that brokers, for a small reward, advise farmers on which weaner is likely to grow faster, determined subjectively on a perceived capacity of a weaner to “chew,” since it will be mainly fed on crop residues.

Small-scale farmers that sell piglets or young pigs for fattening typically sell direct to other farmers, while older pigs are taken to live pig markets if available. Pigs are also sold direct to brokers—in all places were interviews were conducted, farmers reported that they know who to contact, if the need to sell their pig arises. Backyard slaughter at farm or restaurant level was reported and observed in this type of farming. A proportion of live pigs that are bought by brokers are transported to local bars and restaurants for slaughter. At farm level, slaughter activities occur occasionally, especially when a pig seems weak and no buyer wants it, or in case of rejection from market (and low price offer). When on-farm slaughter occurs, a veterinarian is usually called to carry out inspection, with however no record left behind for verification and traceability.

Veterinary inputs for small-scale farmers are limited, but farmers may buy drugs (antibiotics or anthelmintics) direct from agro-vet stores, or consult paraveterinarians. Use of herbal remedies is widespread.

##### Medium Scale Pig Production

Medium-scale pig production farms in Rwanda had between 3 and 30 grown breeding pigs. Medium pig keepers are found in all regions of Rwanda. However, farms of this type are located primarily on the outskirts of main towns and as a novel method of income generation in regions where smallholders typically do not rear pigs. Farmers in this category could be grouped into three types: (1) Farmers operating as a cooperative that often has received pigs as a donation from the government or non-governmental organisations (NGOs), (2) Farmers who also grow crops and run pig farming as an additional source of manure and income, and (3) Persons with another primary employment who engage in pig farming to make money and operate farms through employees. However, farms owned by cooperatives and those operated remotely through employees were found to have shorter lifespan. Cooperative members often join to get a weaner of their own and then leave the cooperative, and the third category fails because of poor supervision of activities at farm level.

Medium-scale farmers mainly sourced weaners or a few sows from their counterpart medium-scale farmers or from large farms that also operate as pig breeders. The pigs in this system are usually confined in pens with a cement floor, walls made of bricks or timber, and a roof made of sheet metal. However, piglets were frequently seen roaming inside the farm. Participants reported that medium scale farmers feed their pigs on cereal bran (mainly maize and rice depending on local availability), supplemented with various crop residues and kitchen/restaurant leftovers. The restaurant leftovers were acquired for free (in places with low demand for leftovers) or by payment [average RWF 25,000 (USD 26) per month]. Grass was also given either between two cereal meals or in the afternoon/evening when pigs are cereal-fed once a day.

Farms of this size are characterised by few or no veterinary inputs, however some reported calling a private veterinary paraprofessional or sector veterinarian when there is suspected disease on the farm or in case of on-farm slaughter activities. Some farms had a veterinary paraprofessional hired to work as farm manager and ensure proper feeding and monitor pig health. In some of the farms belonging to this category, we have identified the presence of veterinary drugs like antibiotics and vitamins at farm level which were reportedly used by pig keepers or veterinarians called for interventions.

Manure produced at these farms is often utilised in the owner's crop fields as fertiliser and pigs from this category are mainly bought by brokers on behalf of slaughterhouses. In addition, on-farm slaughter is often carried out and meat is supplied to bars in towns or in some instances to processing factories.

##### Large Farms

Large farms had between 40 and 800 pigs. There are fewer than 100 large farms in the country, which are located mainly in and around Kigali, with some in other regions, namely Nyagatare, Rubavu, Rulindo, and Gicumbi Districts. Around 60% of large farms had 40–100 grown pigs, and 40% had >100 grown pigs. Pigs at this type of farm are kept in well-constructed pens and are rarely allowed to roam outside the pen. The farmers in this category engage in this activity as a perceived emerging business that can generate high income; all these farms grow crops and are sometimes involved in production of other livestock species. These farmers focus much of their time on this business and unlike medium-scale farmers, they mostly do not have other employment. Some of the farms in this category are owned by larger organisations and in such cases, daily management of pig farming may be delegated to employees, but the supervision is stricter and more regular, as the business is integral to the institution's activities. In addition, farmers reported that they consider manure from pigs as having superior quality compared to manure from cattle, thus very beneficial for their crop fields.

Large-scale farmers started the business with weaners and/or sows bought from counterpart large farms, some of which are pig breeders. However, as the business grows, they breed their own weaners to sustain production. A few farms reported that they import sows and/or boars from Europe or practised artificial insemination using semen collected from their own imported boars, to diversify their farm breeds.

On large farms, pigs are mostly fed on farm-made ration composed of various market-acquired raw ingredients such as maize grains, soya, or cotton seed cake, sprats (*Limnothrissa miodon*) from local lakes, vitamins, and minerals. Making a farm ration is perceived as a measure to reduce costs and to ensure feed quality. Some large scale pig keepers were using commercial feed from local manufacturers, especially for weaners.

Grown pigs produced from large farms are sold to pork traders who transport the live pigs to slaughterhouses located in Kigali, northern and north-west parts of the country. In addition, these farms carry out on-farm slaughter in order to supply pig carcasses to processing factories, usually under veterinary supervision.

Most of the very large farms have a veterinarian (or veterinary paraprofessional) who monitors the health of pigs at the farm and offers treatment when needed. However, two large farms reported that they treated pigs themselves using drugs bought in agro-vet shops, as they did not trust the quality of veterinarians operating in the region.

#### Other Main Actors in the Pig Value Chain

Additional actors were identified as key to pig production and pig sales transactions. These are NGOs, brokers (or middlemen) and pig traders, and veterinarians.

##### Non-Governmental Organisations

Non-Governmental Organisations are involved in providing pig donations to vulnerable rural people. In addition, the NGOs provide training related to animal production and health, especially to beneficiaries of their donations.

##### Brokers

Brokers are paid to buy on behalf of another chain actor (mainly traders and/or slaughter slabs and sometimes farmers). In some occasion, brokers were given money in advance, in order to be able to buy a higher number of pigs for the traders or slaughter slabs.

##### Traders

Traders, small, or large depending on volume of sales, carry out transactions for their own profit. However, there is no barrier between brokers and small traders, as brokers were found to play both roles when needed.

At farm level, brokers sometimes intervened in transactions between small scale farmers who trust their expertise in bargaining. In addition, it was reported that brokers were solicited when a large number of weaners were needed by NGOs for donations. In this case, weaners are bought from rural farmers and pig markets by brokers on behalf of a pig trader contracted by the NGO.

### Chain Profiles for Live Pig Markets

#### Source of Pigs

Live pig markets are mainly located in Southern and Western provinces of Rwanda (Figure 1). It has been estimated that 170,000 pigs per year are sold in Southern province markets, 140,000 per year in Western Province, only 1,400 per year in Northern Province and none in Eastern province (2). Southern province markets are supplied with pigs mainly by farmers (those who have raised the pig for at least 1 month or more) (Figure 4). The remaining pigs are supplied to markets by brokers, who buy pigs in rural areas or smaller livestock markets and re-sell in the market for a slight profit of up to RWF 2000 (USD 2.30) per pig. Small pig traders reported that they may occasionally keep a pig bought from farmers or market to fatten it before resale.

Markets in western Rwanda are mainly supplied by pig traders who bring pigs bought from Southern province markets. In those markets, a smaller proportion is provided by local farmers and the few local brokers that source pigs in nearby areas.

According to interviewees farmers prefer selling and buying from markets because of a wide choice. In addition, the gathering of many pigs allows “standardisation” of the pig price thus setting a fair basis for bargaining. Brokers and traders were reported to prefer buying pigs from markets because of the large range of options offered by a market compared to buying from individual farms. In addition, markets allow them to buy the required number of pigs at one time without having to visit many pig farmers, thus increasing efficiency.

#### Selling of Pigs

Typical prices at market for weaned piglets of about 2–3 months in 2018 were RWF 8,000–12,000 (USD 8.40–10.50) while medium-sized pigs of about 8–12 months (live weight 40–70 kg) were sold for RWF 38,000–65,000 (USD 40–70). Larger pigs (>100 kg) can cost RWF 100,000 and above (USD 105 and above) depending on markets and seasons. After selling, the buyer pays a market tax of RWF 500 (USD 0.53).

In markets, there is a grading system for weaners and young pigs, based on the colour of the skin. White pigs (locally termed “*igitare*” for white), followed by pigs with mixed white and black skin (“*urubayi*”) are perceived as better breeds and get higher prices. These lighter pigs are more likely to originate from exotic breeds with higher growth performance. Black (“*umukara*”) and mixed colour coats (“*urunyombyi* “or “*igisoro*”) attract lower prices, with the mixed colour coat generally perceived as a very bad choice. For grown pigs, this system is less useful and was not used. Instead, live pigs are evaluated on weight, perceived ratio of meat-fat and overall size. Because of the lack of capital and/or improper cold chain at local butcheries and restaurants, it was reported that pork sellers preferred a medium-sized pig of 50–70 kg which can be slaughtered and sold off before the meat spoils. Larger pigs (locally known as “itoni” for tonne) in some regions, are reported to be bought only by traders and brokers that transport pigs to western slaughterhouses, markets, and/or directly for export of live pigs to the Democratic Republic of Congo (DRC).

#### Transport of Pigs

The majority of pigs going to markets were reported to be trekked by farmers to the market. Some brokers also were reported to use this method to bring 1–5 pigs to the market on a journey that could take up to an overnight trek (a maximum of 30 km). A few farmers and brokers reported using a bicycle or motorbike to transport a pig. Similarly, after selling, most pigs were taken on foot to farms or slaughter slabs, or in few cases on a bicycle or motorbike. However, traders buying many pigs for sale in Western province and neighbouring DRC use trucks to transport pigs, usually with other species such as goats and sheep in the same truck.

In western Rwanda, in Karongi and Nyamasheke Districts, live pigs are taken across the border for sale in neighbouring DRC. The number of live pigs exported each year is unclear, but it has been estimated that around 70% of all livestock sold at market in Rwanda is exported to DRC, with at least 93,000 live pigs exported per year in 2015–16, though the methodology behind these estimates is not clear (2). Transport was organised through specific cooperatives: one was reported to use trucks to transport pigs from markets to the shores of Lake Kivu, and another cooperative was in charge of loading pigs into a boat to cross the border through the lake.

### Chain Profiles for Pig Slaughterhouses

#### Characteristics of Pig Slaughterhouses

We identified seven slaughter slabs operating on a regular basis, and one modern slaughterhouse in Kigali. The seven slaughter places are located in Western province (three in Rubavu, one in Rusizi), one in Southern province (Gisagara), and two in Northern province (one in Musanze and one in Gakenke) (Figure 1). We were informed of other slaughter slabs mainly in southern province operating irregularly when they receive orders from restaurants and/or meat processing factories.

The Kigali slaughterhouse operated every day and slaughtered on average 110 pigs per day (range 85–140 pigs). The slabs slaughter between 50 and 200 pigs a week, with those located in the west operating every day (slaughtering 30–40 pigs a day) and those in north Rwanda slaughtering 15–25 pigs a day, three times a week on average. These numbers may be underestimates, but they suggest that the vast majority of pork consumed in the country is slaughtered informally, not at slaughter slabs. The slaughter slab in the South is owned by a local authority, operates on market day and slaughters only 2–4 pigs for local consumption. At this slab, the meat trader was the main actor who organises the slaughtering of pigs and the selling of pork and by-products, with all meat sold on the same day. In some instances, it was found that a pig slaughter slab operated to order from butcheries in Huye town or pork processing factories in Kigali. In that case, the slaughtering is organised by brokers who buy live pigs from farmers and/or market and organise the slaughtering and transport of the carcasses.

In slaughter slabs located in northern Rwanda, the slaughter facilities are privately owned, either by cooperatives or an individual owner. Here, the slaughtering is organised by the slaughter slab itself which mostly operates to orders from pork processing companies based in Kigali or butcheries which have retail shops at the border with DRC. In a few cases it was reported that these slaughter slabs received orders directly from customers in DRC. These slaughter slabs are also used by local pork traders for pork supply, in addition to pork from backyard slaughter. Slaughter slabs in Western province operate in the same way as those in Northern Rwanda. However, their target market is mainly DRC. The slaughter slabs and the slaughterhouse are attended by a veterinarian or a paraveterinarian who inspect the slaughtered pigs according to national adopted guidelines from the FAO (27), except that ante-mortem was not done in any surveyed abattoir.

In addition to slaughter slabs and on-farm slaughter in some large farms, there is a large amount of “backyard” slaughter for local consumption. In many areas of Rwanda, whether a slaughter slab is available or not, the meat traders were key in organising backyard slaughter of pigs and the supply of meat to places selling fried pork (bar/restaurant/hotel). We found that most places practising backyard slaughter were known and visited by local veterinary meat inspectors. However, the inspection was not regular and no records were kept.

#### Source of Pigs

Live pig markets were identified by slaughterhouse managers as the main source of animals for slaughter and pig traders were reported to be the focal suppliers of pigs into the slaughter slabs (Figure 5). One abattoir was supplied with live pigs by pig traders who owned the abattoir and operated as a cooperative. The supplied pigs are mainly bought from southern markets located at a distance of 130–170 km from the abattoir.

However, due to lack of live pig markets, slaughter slabs in northern Rwanda were reported to use a network of brokers who source pigs amongst rural farmers and transport them to the slaughter slabs. In this case, some brokers in need of cash could seek an advance payment from the slaughterhouse. In northern abattoirs, brokers reported that they source pigs mainly from nearby areas but occasionally from as far as western Uganda. Pigs were usually trekked to the slaughter slab, sometimes for long distances, or brought tied on bicycles. For the Kigali slaughterhouse, pigs were transported in trucks by pig traders, mainly from southern markets and northern farms. For western abattoirs, pigs to be slaughtered were transported by pig traders using trucks from distances of up to 200 km.

#### Slaughtering and Sale of Pig Carcasses and Offal

With the exception of the sole modern slaughterhouse, the other seven operate from basic infrastructures, with poor hygiene (see section Sanitary Risk Practices), though equipped with a roof and a concrete floor as recommended (28, 29).

Slaughter slabs were identified as the main source of pork for export and for local pork processing factories. Only a small proportion of pork from slaughter slabs is consumed locally which leaves the local consumer to source their pork from dealers operating informally through backyard slaughtering. Thus, slaughter slabs in Western province slaughter almost exclusively (99%) for export to DRC. Whereas, slaughter slabs in Northern Province operate to order from pork processing companies based in Kigali. Offal such as liver and heart was sold locally for human consumption whereas intestines and lungs were sold to be fed to pigs or dogs.

### Chain Profiles for Processing Factories

We identified five processing factories, four of which are located in Kigali and one in Rulindo, Northern Rwanda (Figure 1). Four of the processing factories are supplied by slaughter slabs (80%) or farm-level slaughtered pigs (20%) (Figure 6). The other processing factory is run jointly with the modern abattoir.

These plants process 30–60 carcasses per week into various products (meat cuts, hams, bacon, etc.) which are sold in shops and/or supermarkets owned by the processing factories, supplied to hotels and other supermarkets in Kigali or supplied to customers in eastern DRC.

Transportation of slaughtered pigs was organised by the slaughterhouse managers or cooperative using vans designed for that purpose. Some processing factories were found to organise transport of pork from slaughter places, using refrigerated vans.

All processing factories mapped had a retailing butchery shop and or supermarket outlets located in Kigali or on the north-western border with DRC. These outlets were reported as the main destination (estimated at 60% of the production) of pork products sold by the factories (fresh meat cuts, sausages, ham, etc.). In addition, these factories were reported to supply hotels (25% of the production) and other supermarkets in Kigali (15%).

### Governance Themes

#### Dominance, Market Information, Technical Knowledge

Our results demonstrate the dominance of smallholders and brokers in the pig value chain. Smallholders, with less than three grown pigs, are dominant in the supply of pigs for markets and slaughter slabs, though the number of large farms is increasing. Brokers and middlemen [also called in Kinyarwanda “abacayi” or “abasherisheri,” from the French “chercheurs” (searchers)] dominate deals of live pigs and facilitate negotiations between small farmers and pig traders (sections Typology of Pig Farming Systems in Rwanda and Other Main Actors in the Pig Value Chain). Brokers act as middlemen and sometimes as resellers buying from small holder farmers and reselling either to butchers or pig traders. Brokers play a key role in live markets by linking farmers to pig or pork traders, and where tongue palpation is practised, brokers are in charge of performing the palpation and declare a pig infested or not. Middlemen sometimes are resellers. Finally, brokers are used by slaughter slabs and processing factories to search for pigs in rural area. Pig traders, who transport pigs in western parts of Rwanda and/or neighbouring DRC, dominate the markets in terms of setting prices and deciding on the number of pigs to be bought.

In rural areas where the majority of small-scale pig farmers reside, the supply of veterinary drugs and services was dominated by private veterinary paraprofessionals operating sometimes under coordination of the local public veterinarian (usually a sector veterinarian).

#### Rules and Incentives

Starting a pig farm does not require any formal permission. Large farms must be situated away from urban areas and pigs in all farm categories have to be kept in pens. Farmers in most of the country do keep pigs in confinement but may let them roam within the household, especially piglets or when they need to remove manure. However, many smallholders in the southern part of Southern Province allow pigs to freely graze outside the household, though this practise can cause a farmer to be fined, based on the Ministerial Order on stray cattle and other domestic animals (19).

Formal rules in live pig markets are limited to the payment of a tax for each pig purchased. In addition, if pigs are to be transported from one District to another, a transport permit is needed, issued by local government veterinarians at the market. Informal rules in live pig markets are set by brokers, and include the need for a tongue palpation before purchase (see next section). The value of a pig to be bought is determined by its size but weaners are valued in some places according to the colour of their coat as an indicator of how fast they will grow.

In slaughter slabs, pigs were supplied by brokers without any form of formal contracts. The abattoir organises inspection of slaughtered pigs (see next section) by a veterinarian (public or private). In addition, there is a fee paid by the pork seller for using the slaughter slab in addition to taxes. According to Rwandan regulations, anyone who wishes to become a butcher must obtain written authorisation from the district authority. If there is no available public slaughterhouse, permission must be sought from the administrative authority in order to allow slaughtering outside formal settings. Anyone who needs to slaughter animals without having a permit to practise butchery, must apply for permission at administrative sector level and specify the time and venue for the slaughter activities. In all cases, meat inspection must be carried out by veterinarians and a monthly report of all slaughtered animals must be sent to the district authority. Visited abattoirs had authorisations from the local administrations and inspections of pig carcasses were carried out by state veterinarians or veterinarians privately working at the facility.

#### Challenges and Business Barriers

It was reported that feed provision constitutes the largest challenge for pig farming. Medium and large scale pig farmers perceive the price of raw ingredients to be high. While small holders reported that it was difficult to get crop residues during dry season when crops are scarce. All sizes of farm in the sector therefore face challenges accessing feed.

The occurrence of a seasonal disease was highlighted by farmers and brokers in various regions. This disease was associated with the death of many pigs during the long dry season (June to September). It is likely to be swine erysipelas according to veterinarians, though some farmers considered it to be African swine fever. However, in past years, both diseases have been detected and reported to the World Organisation for Animal Health by Rwanda Veterinary Services.

Medium and large farms reported lack of abattoirs as one of the main challenges which prevent them from selling added value products (meat cuts) at higher prices instead of live pigs. The few abattoirs locally available are of poor quality, mostly in old buildings. These slaughter slabs only operate when they have orders from pork processing companies in Kigali or Rubavu or orders from DRC. These farms also reported lack of breeding stock which according to them, can lead to decrease in the pig genetic performance caused by inbreeding.

Brokers identified the lack of capital as their main challenge, preventing them from buying more pigs for more profit.

Public veterinarians reported that they did not have enough time for small stock animals due to priority given to cattle farming. Veterinary services were reported to be performed by veterinary paraprofessionals, who sometimes also own veterinary pharmacies.

### Sanitary Risk Practises

#### Farms

Veterinary inputs for small-scale farmers are limited: farmers perceived prices of veterinary drugs and services as high and use traditional medicines (herbs such as *Vernonia amygdalina* and *Tetradenia riparia*, locally known as *umubirizi* and *umuravumba*, respectively) as anthelmintics. However, it was reported that some rural farmers bought anthelmintics from local veterinary pharmacies when they thought that a piglet was not growing fast enough or does not have appetite, and after failure of herb treatment.

Medium and large pig farms were reported to have a private veterinary paraprofessional or veterinarian who visits the farm on a regular basis, thus moving from one farm to another. The veterinary paraprofessional or veterinarian may also be employed as farm manager to take care of pig health matters. In these types of farm, farmers also tend to have their own stock of veterinary drugs and often treat pigs themselves. These types of farm do carry out slaughter at times on the farm. It was reported that in this case, a veterinarian or veterinary paraprofessional is called for inspection.

All sizes of farm had biosecurity flaws that expose pigs to various infectious diseases such as African swine fever and cysticercosis. There was a lack of fences in many small, and some medium, farms which means pigs can roam in and around the households. In addition, introduction of new pigs to the farms is not preceded by quarantine. Quarantine was not reported at any size of farm, including large farms, and we saw no evidence of quarantine facilities. Lastly, farms of all types are frequently visited, by brokers and paraveterinarians with no disinfection measures. Such visitors may be a source of disease spread as they also visit other farms, live pigs markets, and slaughterhouses.

#### Veterinarians

Veterinarians with various qualifications intervene in pig farming as suppliers of veterinary inputs and services. These include fully qualified veterinarians, animal scientists and paraveterinarians with advanced diploma or high school certificate in veterinary sciences. These veterinarians may own veterinary pharmacies (also known as agro-vet shops as they also sell agricultural inputs) where farmers buy drugs, especially anthelmintics, for administration to their pigs. Local veterinarians or veterinary paraprofessionals were reported to buy anthelmintics, vitamins, and antibiotics from these pharmacies to use during their services in various farms.

#### Markets

At pig markets, there was no formal veterinary inspection or hygiene measures. Pigs were systematically tongue palpated by brokers to check if they have larval tapeworm cysts, which if present, would indicate that pigs have cysticercosis and may transmit infection to humans. Interviewees reported that cyst-positive adult pigs lose value and are bought at a lower price by traders for export to DRC, citing looser meat inspection mechanisms at destination. Each cyst found was reported to cause an average of RWF 5,000 (USD 5.50) discount off the initially agreed price, thus if only three cysts were found, the initial price would be reduced by RWF 15,000 (USD 16.5), representing 20–40% lost value for a medium-sized pig (40–70 kg live weight). It was also reported that a cyst-positive weaner would not be bought by local farmers as it is perceived to not be capable of growing well. If pig sellers are not happy with the discounted price, respondents reported that they may take it back to the village and either remove cysts from the tongue using a pin and try to sell it again, or convince neighbours to backyard slaughter it and sell the pork for household consumption. Pigs were not checked for other diseases, however an injured, weak or perceived sick animal was reportedly bought at lower price for slaughter.

It was reported that any pig not growing well and not responding well to treatment was taken to the market to avoid it dying and causing complete loss of money to the farmer. At farm or market, when an animal seems weak, it is bought by brokers, and is slaughtered. A veterinary paraprofessional or veterinarian is called upon to perform inspection without ante-mortem inspection. A condemned pig is buried in the presence of the veterinary inspector but it was reported in several FGDs that sometimes buried pigs are unearthed after departure of the veterinarian and they are consumed. To dissuade people from eating the condemned meat, inspectors were reported to sometimes spray fuel oil on the carcass before burial.

#### Slaughter Slabs

Apart from the sole modern abattoir, we observed during visits that sanitary conditions at the seven slaughter slabs were poor. Killing and bleeding (defined as dirty operations) and evisceration and carcass splitting (clean operations) were not usually conducted in separate zones. In addition, pig scalding to remove hair involved using the same barrel containing hot water for all pigs slaughtered the same day. Carcasses were prepared on the floor and no running water was available inside the seven slaughter slabs.

At slaughter slabs, there is regular veterinary inspection and the veterinarian (or veterinary paraprofessional) inspects the carcass of every pig slaughtered but does not inspect offal. The veterinarian will assess the overall appearance of the carcasses for indication of disease and will specifically make cuts through specific organs and the carcass as per the FAO meat inspection recommendations (27). This professional decides whether or not to pass the pork for human consumption. However, there were no veterinary records of inspected pigs at any slaughter slab visited.

We observed divergence of practise on the decision taken for a cysticercosis carcass. In northern parts of Rwanda where cysticercosis is rarely seen according to veterinarians, a pig found with cysts was reported to be condemned totally (whole carcass condemnation). However, in southern Rwanda, where cysticercosis is more prevalent, it was reported that if a pig does not have a significant disseminated infestation, only the affected organs would be condemned and the veterinary inspector would advise to cook pork thoroughly. There was no ante-mortem inspection carried out at any of the visited slaughter slabs.

In case of pig slaughter at a farm or restaurant backyard, a private veterinary paraprofessional or veterinarian is usually called to undertake the inspection and give permission for pork consumption.

## Discussion

This value chain study was conducted in order to define the structure and governance of the pork value chains in Rwanda, and deduce vulnerabilities and safety risks associated with its functionalities. Overall, the Rwandan pig industry is at the early stages of transition with an increasing number of emerging modern farms. The rapid increase in demand for pork meat is met largely by smallholder farmers who lack institutional support and veterinary oversight. Where large farms operate, they do so without a structured market for inputs or for selling their production. Pig production offers income generation opportunities to all actors in the value chain. Particularly, it is hailed by smallholders who prefer it for its quick returns and for manure used in crop production. There are opportunities for growth, such as a better organisation of pig marketing, an increase in the number and quality of pig slaughterhouses, improved supply of breeding stock and quality feed. Given the current operations of the veterinary services, the progress registered in the Rwanda pig industry may be challenged by highly contagious diseases such as African swine fever and swine erysipelas and prevalence of zoonotic diseases in the food chain.

To the best of our knowledge, this is the first comprehensive analysis of the pig value chain in Rwanda. Our data show the existence of three types of pig production in the country, consistent with the findings of Mbuza et al. (4) in their assessment of the status of semi-intensive and intensive pig production systems. The pig production system in Rwanda is dominated by smallholders raising one to two grown sows, who are located in rural areas and run pig production as an activity complementing crop production. These types of farms are linked to pork consumers and pig processing factories including slaughterhouses through brokers and live pig markets, and rely on brokers to make deals. While raising pigs is seen as a profitable business for small-scale farmers who invest little in their pigs, the lack of biosecurity and limited veterinary services expose the pigs to diseases and thus may weaken food security.

Medium and large pig farms constitute an emerging alternative source of pigs in Rwanda, contributing to the supply of pork with better quality pigs. However, due to the high cost of feed, many of the medium-sized farms reported that they did not make significant profits. Feed challenges were also highlighted by Mbuza et al. (4), and this is a consistent message from other countries in the region (18, 30, 31). The high price is reported to be associated with a lack of raw materials on the local market due to direct competition with markets for human food, and with costs associated with import of feed ingredients from neighbouring countries. While the Rwandan government is promoting the investment and establishment of feed mills as a strategy to increase availability of livestock feed, efforts should also focus on investigating alternative, cheap and innovative source of animal feeds such as insects (17) as well as a better integration of crop and livestock to optimise mutual benefits between agriculture and livestock production (32, 33).

Transactions involving pigs outside market structures were done between farmers of comparable farm size located in the same vicinity. This may constitute a challenge for current ambitions to expand pig production in Rwanda. The fact that pigs are exchanged between neighbouring farmers with poor or no traceability increases the chances of inbreeding which may result in poor performance of offspring. Indeed, we saw that piglets with “exotic” genetics were more highly valued in the market, attracting higher retail sale prices. The pig breeding system in Rwanda is inadequate (4), with the choice of stock found to be influenced by breed availability in nearby farms (34.1%), advice from other farmers (65.4%), or donation opportunities (0.5%), in line with our observations.

Live pig markets were reported by all value chain actors to be venues of choice enabling more options for sellers, allowing price standardisation for sellers and buyers and easing collection of large numbers of pigs at the same time for big traders. These live markets allow smallholders to indirectly have access to national and export markets for pigs, with brokers and traders as intermediaries. Pig brokers are key persons connecting farmers to various actors in the chain. The brokers are considered to be knowledgeable, from whom farmers seek advice or in whom they place their trust for making deals. This is even more relevant in places where live pig markets are not available, and where the farmers are left to sell their pigs at the farm-gate to the brokers. This is clearly an influential group of actors in the chain.

The supply of slaughtered pigs relies on few slaughterhouses. A striking finding was the lack of sufficient number of slaughterhouses to satisfy the supply of local pork consumption and ensure proper meat hygiene. In addition, we observed poor hygienic conditions in all visited slaughtering places. These abattoirs are set up to mainly serve pork processing factories and pork export to DRC and high-end consumers in supermarkets and hotels, thus leaving other pork traders to source their supply from informal slaughtering. There were also significant on-farm slaughtering activities. The majority of interviewees reported that it was the result of the lack of pig abattoirs and efforts to sell more profitable added-value products. Conversely, some small famers reported that the abattoir fee was a barrier, resulting in more informal slaughter.

Veterinary services were characterised by a very limited availability of qualified veterinarians and a greater presence of veterinary paraprofessionals, though numbers of these are also limited. In addition, public veterinarians focused their services on cattle production, leaving other livestock production systems to be largely dependent on paraveterinarians. Thus, there is very little competition between veterinarians and paraveterinarians for the provision of services to pig farmers. However, paraveterinarians lack a proper reporting system and an adequate supervision mechanism. As a result, there is very limited information on the prevalence of potentially important pig diseases in small holder production. The findings of the last performance evaluation of Rwanda veterinary services by the World Organisation for Animal Health (OIE) highlighted poor management and supervision of paraveterinarians by veterinarians (34). There is a clear need to ensure that veterinary paraprofessionals are well-trained and equipped with skills that enable them to carry out activities related to animal health and veterinary public health. In addition, their activities must be placed under the overall supervision and responsibility of veterinarians (35). Moreover, for reasons of cost, small-scale farmers often rely on traditional treatment using herbal medicines. The plants used have also been used in other countries and their traditional efficacy as anthelmintics and antimicrobials is recognised (36–38).

The observed lack of biosecurity measures at farms, and insufficient confinement of pigs, may be a vehicle for disease introduction and circulation from the environment and from other pigs (39). In particular, the lack of quarantine for new pigs and the lack of disinfection for visitors is a concern in farms of all sizes. This low level of biosecurity and lack of veterinary services is typical for the region (31, 40–42). Medium and large-scale pig farmers largely confined pigs in pens, but still had few or no biosecurity measures, which is a known risk factor for the occurrence of African swine fever (43).

Important deficiencies were identified with respect to the potential for food-borne diseases, such as *Taenia solium* cysticercosis, salmonellosis, trichinellosis, brucellosis, and leptospirosis. There is a paucity of information on animal disease prevalence in Rwanda. However, a high prevalence of porcine and human cysticercosis has been reported (44–46), as well as non-typhoidal *Salmonella* isolates which were recovered from pig faecal samples (47). In neighbouring countries, trichinellosis, salmonellosis, and cysticercosis have been documented in pigs (48–52) and in humans (53, 54).With the exception of the single modern abattoir, slaughter houses had intercrossing of clean and dirty operations, from killing through to carcass preparation. This is likely to lead to cross-contamination with pathogens such as *Salmonella* (55), especially as no running water was available inside the visited slaughterhouses, as has been reported elsewhere (49). Installing cold and hot water, as per international guidelines, can help to reduce occurrence of such contamination. The high dependence of local consumption on pork from backyard slaughtering (at bar or farm level) also poses a food safety concern. Informal slaughtering has been reported in other countries (7) and lack of adequate regulations is known to contribute to illegal trading and potential introduction of unsafe meat in the food chain (56). In many instances, bars and farms practising backyard slaughter are well-known by local veterinary paraprofessionals and veterinarians and it was reported that meat inspection was carried out upon slaughter. However, we have not been able to verify inspection records at any level. In the absence of records, it is possible that the data are biassed as informants may exaggerate the extent to which inspection is carried out at informal slaughter. Even in visited registered slaughter slabs where veterinary inspection is regularly carried out, there were no records kept on the source of animals, number of animals slaughtered, carcasses approved for consumption, organs or carcasses condemned, or destination of slaughtered animals. Abattoirs are recognised as places where passive disease surveillance can be organised, and have been recognised as a potentially useful tool for the surveillance of diseases including those of zoonotic nature (16, 23, 57, 58). However, the lack of records in Rwandan abattoirs suggests that such surveillance is not currently possible.

Known risk factors for porcine cysticercosis, such as allowing pigs to roam and poor sanitation, were widespread in smallholder production. However, awareness of cysticercosis is also widespread, and it is noteworthy that tongue palpation was regularly practised in southern and western Rwanda. While tongue palpation has value in understanding broad level patterns of infection at the population level (59), it is known to have low sensitivity in identifying individual infected pigs, unless they are heavily infected (60, 61). Moreover, it was reported that positive pigs can still be sold at a lower price, suggesting that tongue-positive pigs can still enter the food chain through alternative routes. While farmers reported that being cheated by brokers was unlikely (e.g., where a broker might falsify relevant details like whether a pig has a cyst on the tongue), this ability to detect cysts can give an advantage to brokers in setting a discounted price for any positive pig. Other studies have described the role of traders and brokers who are capable of organising themselves in such way that benefits go to traders and brokers but not necessarily farmers (8, 13, 14). Either way, it is clear that smallholder farmers can lose significant income due to cysticercosis, so are likely to be willing to engage with control efforts. The risk of cysticercosis can be further reduced by carcass inspection at slaughter, though this still has a low sensitivity.

In conclusion, to our knowledge the current study is the first to analyze the pig value chain in Rwanda, to understand its structure, functionalities, governance, and associated public health risks. Small holders constitute the majority of pig production systems in the country. Pigs provide a valuable source of additional household income, but income is limited by scarcity of feed, heavy reliance on brokers for pig transactions, and lack of veterinary inputs. Our study showed that transactions of live and slaughtered pigs were dominated by a few actors who organise the market, thus potentially penalising farmers and consumers. There are widespread sanitary risks with consequences for both animal and human health.

In order to improve the profitability and safety of the sector, investment is needed to increase accessibility to cheap and diverse feed, and to strengthen breeding and veterinary services. Infection prevention and control programmes should be articulated to help control both epizootic and zoonotic diseases. Last but not least, the sector should be supported by an organisation for pig marketing and by the construction of more slaughterhouses designed to ensure meat hygiene.

There are some limitations to our results, as a consequence of the non-systematic sampling approach as well as the thematic analyses used. Further research studies with more detailed quantitative data will provide additional insights.

This study provides a framework to understand dynamic factors within the Rwanda pig value chains. Future studies should focus on analysing the profitability of various types of pig farms and determine how best to share benefits across the whole value chain. We also recommend further studies to investigate animal welfare conditions throughout the value chain.

## Data Availability Statement

The raw data supporting the conclusions of this article will be made available by the authors, without undue reservation.

## Ethics Statement

The studies involving human participants were reviewed and approved by the Rwanda National Ethics Committee (Reference: No. 165/RNEC/2017) and the University of Leeds Faculty of Biological Sciences Research Ethics Committee (Reference BIOSCI 16-019). The patients/participants provided their written informed consent to participate in this study.

## Author Contributions

AS: collected, analysed data, and drafted the first draft of the manuscript. EF, RQ, AS, and NR: developed the study design and critically reviewed the various manuscript drafts. All authors approved the submitted version.

## Funding

This study was funded by the UK Medical Research Council Global Challenge Research Fund (Grant number MR/P025471/1). EF acknowledges partial support from the CGIAR Research Program on Agriculture for Nutrition and Health (A4NH), led by the International Food Policy Research Institute (IFPRI) and acknowledges the CGIAR Fund Donors (http://www.cgiar.org/funders).

## Conflict of Interest

The authors declare that the research was conducted in the absence of any commercial or financial relationships that could be construed as a potential conflict of interest.

## Publisher's Note

All claims expressed in this article are solely those of the authors and do not necessarily represent those of their affiliated organizations, or those of the publisher, the editors and the reviewers. Any product that may be evaluated in this article, or claim that may be made by its manufacturer, is not guaranteed or endorsed by the publisher.
